# *Artemisia argyi* essential oil modulates lipid metabolism via linoleic acid and glycerophospholipid pathways in high-fat diet-induced obese mice

**DOI:** 10.3389/fnut.2025.1650976

**Published:** 2025-10-01

**Authors:** Kaijun Wang, Anqi Yang, Yunxia Li, Qi Han, Zhangzheng Yin, Siting Xia, Jiayi Chen, Jiahao Mo, Peng Bin

**Affiliations:** ^1^Hunan Provincial Key Laboratory of the Traditional Chinese Medicine Agricultural Biogenomics, Changsha Medical University, Changsha, China; ^2^College of Animal Science and Technology, State Key Laboratory for Conservation and Utilization of Subtropical Agro-Bioresources, Guangxi University, Nanning, China; ^3^Animal Nutritional Genome and Germplasm Innovation Research Center, College of Animal Science and Technology, Hunan Agricultural University, Changsha, China; ^4^State Key Laboratory of Swine and Poultry Breeding Industry, Guangdong Laboratory of Lingnan Modern Agriculture, College of Animal Science, South China Agricultural University, Guangzhou, China

**Keywords:** *Artemisia argyi*, high fat, essential oil, lipid, metabolomics

## Abstract

Obesity is a nutritional disorder caused by an imbalance between energy intake and expenditure. The natural products will provide hypolipidemic active factors that will intervene in obesity and its complications, as well as direct drug treatment. *Artemisia argyi* leaf is a well-known species in traditional Chinese medicine, and its essential oil (AAEO) has been identified to exert various physiological activities. This study investigates the chemical composition of essential oil extracted from *Artemisia argyi* (AAEO) and its potential effects on intestinal microbial function and lipid metabolism in mice subjected to a high-fat diet (HFD). The essential oil was extracted and analyzed for its bioactive compounds, revealing a rich presence of terpenes and oxygenated derivatives. Utilizing LC-MS/MS technology combined with multivariate statistical methods, significant alterations in serum metabolites were observed in AAEO-treated mice compared to those on HFD alone. Key metabolic pathways impacted by AAEO included linoleic acid metabolism and glycerophospholipid metabolism. These findings suggest that while AAEO did not significantly alter gut microbiota composition, it substantially modulated lipid metabolism via linoleic acid and glycerophospholipid pathways, suggesting a potential natural strategy for obesity-related metabolic disorders. This study provides new insights into the application of *Artemisia argyi* essential oil in dietary interventions and obesity management.

## 1 Introduction

The prevalence of obesity and the burden of obesity-related diseases are increasing worldwide. According to the World Health Organization (2023), over 1.9 billion adults worldwide are overweight or obese, with the prevalence continuing to rise (1). Obesity has also become a major public health problem in China. Obesity is characterized by excess adipose tissue, an imbalance between energy intake and expenditure, and is associated with low-grade inflammation and insulin resistance, high fat induced obesity is also associated with atherosclerosis and nonalcoholic fatty liver disease (2–6). Dietary patterns in China have changed significantly compared to the last few decades, and there has been a gradual shift from a traditional plant-based fruit and vegetable diet to a Western-style diet, with an increasing intake of foods of animal origin, refined and processed grains, and foods high in sugar and fat (7). Consumption of ultra-processed foods was found to be associated with the risk of weight gain, overweight and obesity in a prospective study (8). There is also strong evidence that being overweight or obese is associated with an increased risk of major non-communicable diseases (cardiovascular disease, type 2 diabetes and cancer) and premature death in the Chinese population (9–11). Therefore, improving obesity symptoms is imperative and important for intervening in the development of other diseases. Obesity is a complex multifactorial disease. The main causes of its pathogenesis include: environmental factors (mainly physical activity and diet), genetic factors, and gut microbes (12). Some anti-obesity medications, such as orlistat and lorcaserin, as well as phentermine/topiramate and naltrexone/bupropion extended-release tablets have been shown to be effective in reducing weight (13, 14). However, anti-obesity drugs may also lead to gastrointestinal problems, weakness, psychiatric disorders and cardiovascular diseases (15). Therefore, many experts predict that natural products will provide lipid-lowering active factors to intervene in obesity and its complications, as well as direct drug therapy.

Essential oil represents a large family, in which members are almost characterized by volatile and semi-volatile compounds, usually with a low molecular weight. It is generally formed as the secondary metabolite by aromatic plants (16). The chemical composition of essential oil can be categorized by their biosynthetic pathways as terpenes and their oxygenated derivatives (such as alcohols, ethers, aldehydes, ketones, and esters), and some aromatic and aliphatic compounds (16). Essential oil is traditionally obtained by steam or hydro-distillation (17–19). Recently, relatively new techniques, including solid-phase microextraction (SPME) (20), supercritical fluid extraction (SFE) (21), and microwave-assisted extraction (MAE) (22), have been developed and applied to collect these volatile constituents. Essential oil is reported to perform well in alleviating oxidative damage (23, 24), inflammation (25), infection (26, 27).

*Artemisia argyi* is an herbaceous plant distributed in most regions of East Asia. It has been widely used in traditional Chinese medicine for treating eczema, hemorrhage, and dysmenorrhea (28). Dried leaves of this herb are also used in acupuncture clinics in China to treat various diseases, especially chronic conditions such as osteoarthritis, asthma, gastrointestinal disorders, and insomnia (18). *Artemisia argyi* is reported to possess multiple pharmacological activities including anti-inflammatory, antimicrobial, antiasthma, analgesic, antivirus, antioxidant, antitumor and immunomodulatory effects (18, 29–31), most of which are contributed by the essential oil existing in *Artemisia argyi* leaves. *Artemisia argyi* is a rich source of essential oil (AAEO), ~1.13% (w/w) (18). Indeed, the strong and aromatic odors of *Artemisia argyi* are mostly attributed to a high concentration of essential oil. With the help of gas chromatography-mass spectrometry (GC–MS), a total of 33 compounds were initially identified in AAEO, and eucalyptol, also known as 1,8-cineole, was most plentiful, accounting for 23.66% (29). However, the amount of 1,8-cineole was quantified to 33.4% in another study (17). By comparing the phytochemicals of AAEO from eight different areas, locality was considered as a factor affecting the quality of AAEO (32). AAEO has been relatively little studied, although it is a terrestrial herb. The objective of this study was to investigate the chemical composition of AAEO and the effects on gut microbial function and serum metabolic profiles in a mouse model of diet-induced obesity. A systematic study was conducted using ICR mice to determine the alterations in microbial function and serum differential metabolites of AAEO with obesity.

## 2 Materials and methods

### 2.1 Volatile oil obtaining

The volatile oil was isolated by hydrodistillation at the Jiangxi Hairui Natural Plant Co., Ltd. (Jian, China). The leaves of *Artemisiae Argyi* were air-dried before steam distillation. The AAEO after extraction was stored in sealed amber ampules at 4 °C until being tested or analyzed.

### 2.2 GC-MS analysis

The volatile oil was analyzed on a GCMS-QP2010 Plus Mass Spectrometer (Shimadzu) equipped with a DB-5 ms capillary column (30.0 m × 0.25 mm; film thickness, 0.25 μm) and mass spectrometry MS detector (MS). GC conditions were the same as described above), and data were recorded in the full-scan acquisition mode with the range of m/z 45–500. The compounds were identified by comparing retention indices (RI) and mass spectra with data from the NIST 11.0 MS library and the literature.

### 2.3 Experimental animals

Animal experiments were conducted in accordance with the Hunan Agricultural University Institutional Animal Care and Use Committee (202105). Six-week-old male ICR mice were purchased from SLAC Laboratory Animal Central (Changsha, China). A total of 50 male mice (29.40 ± 1.23 g) were divided into 5 groups at random and housed in polypropylene cages and maintained in standard laboratory conditions of temperature (25 ± 2 °C) and light-dark cycle (12 h/12 h). The diets of mice were fed the control diet (Con), the high-fat diet (HFD), and the HFD-fed mice with the low (LEO, 0.20 ml/kg), medium (MEO, 0.40 ml/kg), and high (HEO, 0.80 ml/kg) doses of AAEO as previous study (33). AAEO dissolved in 4% Tween 80 and administered by gastric administration. Before any treatment, mice were housed for 7 days to allow adaptation. The experiment lasted for 8 weeks. At the end of the experiment the animals were sacrificed under anesthesia.

### 2.4 Fecal collection and sequencing

Prepare 1.5 ml sterile centrifuge tubes (marked animal number in advance), sterile forceps, and transfer the mice to a clean, sterile individual feeding cage after 8 hours on an empty stomach. Pick up fresh stool directly with sterile forceps, avoiding hand contact with the sample. Dispense into marked sterile centrifuge tubes immediately after collection and place kept at −80 °C for examination. The fecal DNA extraction and 16S ribosomal RNA amplification were conducted as previously reported (34, 35). The microbial function prediction analysis process refers to previous studies (36, 37).

### 2.5 Serum metabolite extraction and quality control sample

100 μl serum sample was added to a 1.5 ml centrifuge tube with 400 μl solution [acetonitrile: methanol = 1:1(v:v)] containing 0.02 mg/ml internal standard (L-2-chlorophenylalanine) to extract metabolites. The samples were mixed by vortex for 30 s and low-temperature sonicated for 30 min (5 °C, 40 KHz). The samples were placed at −20 °C for 30 min to precipitate the proteins. Then the samples were centrifuged for 15 min (4 °C, 13,000 g). The supernatant was removed and blown dry under nitrogen. The sample was then re-solubilized with 100 μl solution (acetonitrile: water = 1:1) and extracted by low-temperature ultrasonication for 5 min (5 °C, 40 KHz), followed by centrifugation at 13,000 g and 4 °C for 10 min. The supernatant was transferred to sample vials for LC-MS/MS analysis. As a part of the system conditioning and quality control process, a pooled quality control sample (QC) was prepared by mixing equal volumes of all samples. The QC samples were disposed and tested in the same manner as the analytic samples. It helped to represent the whole sample set, which would be injected at regular intervals (every 5 samples) in order to monitor the stability of the analysis.

### 2.6 Serum metabolomics analysis

The LC-MS/MS analysis of sample was conducted on a Thermo UHPLC-Q Exactive HF-X system equipped with an ACQUITY HSS T3 column (100 mm × 2.1 mm i.d., 1.8 μm; Waters, USA) at Majorbio Bio-Pharm Technology Co. Ltd. (Shanghai, China). The mobile phases consisted of 0.1% formic acid in water: acetonitrile (95:5, v/v) (solvent A) and 0.1% formic acid in acetonitrile: isopropanol:water (47.5:47.5, v/v) (solvent B). The flow rate was 0.40 ml/min and the column temperature were 40 °C. The injection volume was 3 μl. The mass spectrometric data were collected using a Thermo UHPLC-Q Exactive HF-X Mass Spectrometer equipped with an electrospray ionization (ESI) source operating in positive mode and negative mode. The optimal conditions were set as followed: Aux gas heating temperature at 425 °C; Capillary temp at 325 °C; sheath gas flow rate at 50 psi; Aux gas flow rate at 13 psi; ion-spray voltage floating (ISVF) at −3,500 V in negative mode and 3,500 V in positive mode, respectively; Normalized collision energy, 20–40–60 eV rolling for MS/MS. Full MS resolution was 60,000, and MS/MS resolution was 7,500. Data acquisition was performed with the Data Dependent Acquisition (DDA) mode. The detection was carried out over a mass range of 70–1,050 m/z.

### 2.7 Statistical analysis

Statistical analyses between the means of each group were analyzed using one-way ANOVA (one-way analysis of variance) followed by Duncan comparison range tests through SPSS 22.0. The statistical significance level was set at *p* < 0.05. The significance between groups represented by ^*^ was *p* < 0.05, ^**^ represented *p* < 0.01, ^***^ represented *p* < 0.001. For the metabolomics data, the raw data were imported into the Progenesis QI software (Waters Corporation, Milford, USA) for data preprocessing after UHPLC-QE-HFX/MS analyses. Through baseline filtering, peak identification, peak integral, retention time correction, and peak alignment. Then, the data matrix containing sample names, m/z, retention time and peak intensities was exported for further analyses. At the same time, the metabolites were identified by searching database, and the main databases were the HMDB (https://www.hmdb.ca), Metlin (https://metlin.scripps.edu/) and the self-compiled Majorbio Database (MJDB) of Majorbio Biotechnology Co., Ltd. (Shanghai, China). The R package “ropls” (Version 1.6.2) was used to perform principal component analysis (PCA) and orthogonal least partial squares discriminant analysis (OPLS-DA), and 7-cycle interactive validation evaluating the stability of the model. The metabolites with VIP > 1, *p* < 0.05 were determined as significantly different metabolites based on the Variable importance in the projection (VIP) obtained by the OPLS-DA model and the *p*-value generated by student's *t* test.

## 3 Results

### 3.1 Essential oil composition

The chemical composition of AAEO was analyzed by GC/MS as shown in Table 1. Only components with concentrations >0.3% are reported. The obtained results indicated the presence of 25 chemical compounds in the chromatogram, constituting 90.79% of the total components detected. The highest compositions of the compounds were 18.43% of Linalool, followed by Bicyclo[2.2.1] heptan-2-one, 1,7,7-trimethyl-, (1S)- (13.28%), Safroleb (11.96%), D-Limonene (10.86%), Tricyclo[2.2.1.0(2, 6)]heptane, 1,7-dimethyl-7-(4-methyl-3-pentenyl)-, (-)- (6.78%), 3-Cyclohexene-1-methanol, alpha.,4-trimethyl-, (R)- (5.53%), and alpha-Terpineol (5.38%).

**Table 1:** Chemical constituents of AAEO identified by GC-MS.

**Peak no**	** $R_{t}^{1}$ **	**Compound**	**Area%**
1	14.165	alpha-Pinene	1.07
2	15.023	Bicyclo[3.1.0]hexane, 4-methylene-1-(1-methylethyl)-	1.45
3	15.161	beta-Pinene	0.77
4	15.276	beta-Myrcene	1.06
5	16.421	D-Limonene	10.86
6	16.488	Eucalyptol	2.25
7	16.79	gamma-Terpinene	0.46
8	17.05	trans-Linalool oxide (furanoid)-	0.3
9	17.394	Cyclohexene, 3-methyl-6-(1-methylethylidene)-	0.41
10	18.154	Linalool	18.43
11	19.284	Bicyclo[2.2.1]heptan-2-one, 1,7,7-trimethyl-, (1S)-	13.28
12	19.739	endo-Borneol	0.48
13	19.905	3-Cyclohexen-1-ol, 4-methyl-1-(1-methylethyl ethyl)-, (R)-	2.66
14	20.273	3-Cyclohexene-1-methanol, alpha, 4-trimethyl-, (R)-	5.53
15	20.288	alpha-Terpineol	5.38
16	22.86	Safrole	11.96
17	24.422	Phenol, 2-methoxy-3-(2-propenyl)-	1.83
18	24.969	2,6-Octadien-1-ol, 3,7-dimethyl-, acetate, (Z)-	0.33
19	25.408	Copaene	0.88
20	25.949	Methyleugenol	0.78
21	27.231	Tricyclo[2.2.1.0(2,6)]heptane, 1,7-dimethyl-7-(4-methyl-3-pentenyl)-, (-)-	6.78
22	27.586	trans-.alpha.-Bergamotene	1.22
23	28.732	Humulene	0.51
24	29.638	Germacrene D	0.47
25	32.404	1,6,10-Dodecatrien-3-ol, 3,7,11-trimethyl-, (E)-	1.64

^1^*R*_*t*_, retention time (min).

### 3.2 Fecal microbial function prediction

**Figure 1:**
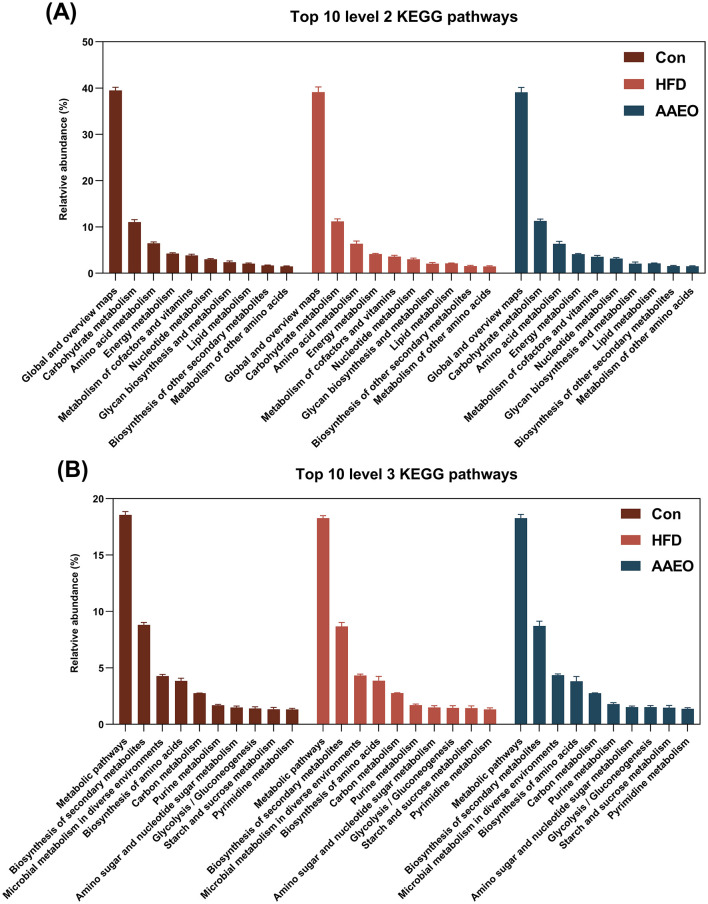
Effects of Artemisia argyi essential oil (AAEO) on top 10 predicted metagenomic functions at level 2 **(A)** and level 3 **(B)** of the KEGG pathways.

As demonstrated in Figure 1, based on PICRUSt prediction, the microbiota function was predicted; the KEGG pathway in top 10 at level 2 about metabolism included global and overview maps, carbohydrate metabolism, amino acid metabolism, energy metabolism, metabolism of cofactors and vitamins, and nucleotide metabolism et al. in three dietary treatments (Figure 1A). Totally, the PICRUSt predicted 295 pathways in level 3 of fecal samples, among them, only 80 pathways were related to metabolism (level 2). The central pathway in top 10 of level 3 contained five pathways about global and overview maps (as shown in Figure 1B), which were metabolic pathways, biosynthesis of secondary metabolites, microbial metabolism in diverse environments, biosynthesis of amino acids, and carbon metabolism respectively. In addition, the pathways of amino sugar and nucleotide sugar metabolism, glycolysis/gluconeogenesis, starch and sucrose metabolism were associated with carbohydrate metabolism. The purine metabolism and pyrimidine metabolism were associated with nucleotide metabolism at level 3. Finally, the top 10 pathways in KEGG levels 2 and 3 were not notably different (*p* > 0.05). Analysis of the AAEO's effects on the mouse fecal microbiota demonstrated that it influenced the functional potentials of these microbes (Table 2). Compared to the Con group, the HFD group with AAEO significantly raised (*p* < 0.05) abundance of lysine biosynthesis, tyrosine metabolism, pyruvate metabolism, and glycerolipid metabolism related with amino acid, carbohydrate, and lipid metabolism. On the contrary, the HFD group with AAEO significantly decreased (*p* < 0.05) abundance of phenylpropanoid biosynthesis, pentose and glucuronate interconversions, oxidative phosphorylation, carbon fixation pathways in prokaryotes, and cyanoamino acid metabolism than Con group. Although HFD significantly increased the abundance of the other glycan degradation pathway associated with glycan biosynthesis and metabolism (*p* < 0.05), the addition of AAEO to HFD had no effect on it (*p* > 0.05).

**Table 2:** KEGG pathways that showed different abundances between fecal microbiota by fed AAEO.

**Level 2**	**Level 3**	**Pathway ID**	**Con^1^**	**HFD^2^**	**AAEO^3^**
Amino Acid Metabolism	Lysine biosynthesis	ko00300	0.56 ± 0.04^b^	0.58 ± 0.05^ab^	0.63 ± 0.06^a^
	Tyrosine metabolism	ko00350	0.17 ± 0.02^b^	0.18 ± 0.02^ab^	0.19 ± 0.01^a^
Biosynthesis of other secondary metabolites	Phenylpropanoid biosynthesis	ko00940	0.17 ± 0.03^a^	0.14 ± 0.03^ab^	0.12 ± 0.05^b^
Carbohydrate metabolism	Pyruvate metabolism	ko00620	1.01 ± 0.04^b^	1.04 ± 0.06^ab^	1.08 ± 0.05^a^
	Pentose and glucuronate interconversions	ko00040	0.38 ± 0.06^a^	0.34 ± 0.09^ab^	0.30 ± 0.06^b^
Energy metabolism	Oxidative phosphorylation	ko00190	1.00 ± 0.09^a^	0.93 ± 0.05^ab^	0.91 ± 0.09^b^
	Carbon fixation pathways in prokaryotes	ko00720	0.93 ± 0.05^a^	0.88 ± 0.05^ab^	0.84 ± 0.08^b^
Glycan Biosynthesis and Metabolism	Other glycan degradation	ko00511	0.49 ± 0.10^a^	0.37 ± 0.07^b^	0.37 ± 0.12^b^
Lipid Metabolism	Glycerolipid metabolism	ko00561	0.36 ± 0.07^b^	0.42 ± 0.05^ab^	0.44 ± 0.06^a^
Metabolism of other amino acids	Cyanoamino acid metabolism	ko00460	0.33 ± 0.03^a^	0.29 ± 0.03^ab^	0.28 ± 0.04^b^

^1^Con, control diet; ^2^HFD, high fat diet; ^3^AAEO, high fat diet with HEO. In the same row, values with no letter or the same letter superscripts mean no significant difference (*p* > 0.05), while with different small letter superscripts mean significant difference (*p* < 0.05).

### 3.3 Data quality control and identified metabolites

**Figure 2:**
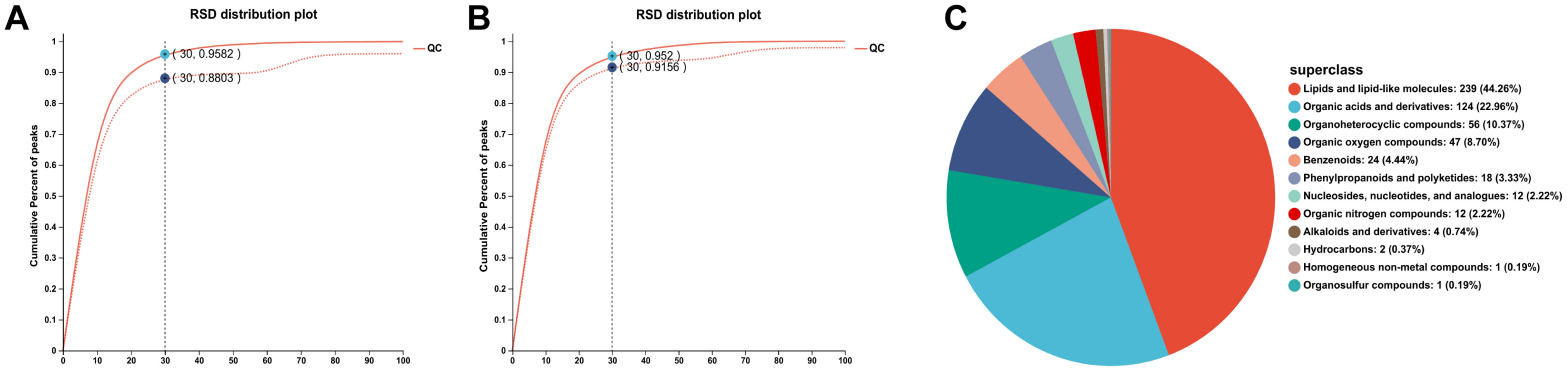
QC sample positive ion evaluation diagram **(A)** and QC sample negative ion evaluation diagram **(B)**, HMDB compound classification **(C)**.

The QC sample positive (Figure 2A) and negative (Figure 2B) ion assessment plots were shown in Figure 2. A QC sample was injected every eight samples to verify the system stability during the whole sample batch. For the overall data, the RSD < 0.3 and the percentage of peak accumulation >70% indicated that the proposed method was stable, and the overall data measured were qualified (dashed line indicates before pretreatment, solid line indicates after pretreatment). It can be seen that when the RSD was 30%, the peak accumulation percentages of positive ions and negative ions of QC samples are 95.8% and 95.2% respectively. It indicated that the data is stable and reliable and can be used for subsequent analysis. The metabolite classification information obtained based on the identification of HMDB database is shown in Figure 2C. It can be proved that the metabolite species of mice blood were mainly concentrated in lipids and lipid-like molecules, organic acids and derivatives, organoheterocyclic compounds, benzenoids, and phenylpropanoids and polyketides. Lipids and lipid-like molecules were the most abundant metabolites with 239, followed by organic acids and their derivatives with 124.

As shown in Table 3, a total of 7,107 positive ion mass spectrometry peaks and 5,106 negative ion mass spectrometry peaks were identified by LC-MS/MS non-targeted metabolomics; 357 positive ion metabolites and 243 negative ion metabolites compared to public databases; and 173 positive ion metabolites and 102 negative ion metabolites compared to KEGG database.

**Table 3:** Total ions and identification statistics chart.

**Ion mode**	**All peaks^3^**	**Identified metabolites**	**Metabolites in library**	**Metabolites in KEGG**
Pos^1^	7107	471	357	173
Neg^2^	5106	314	243	102

^1^Pos, positive ionization mode, ^2^Neg, negative ion mode; ^3^All peaks, number of mass spectral peaks extracted by the software.

### 3.4 PCA and PLS-DA analysis

**Figure 3:**
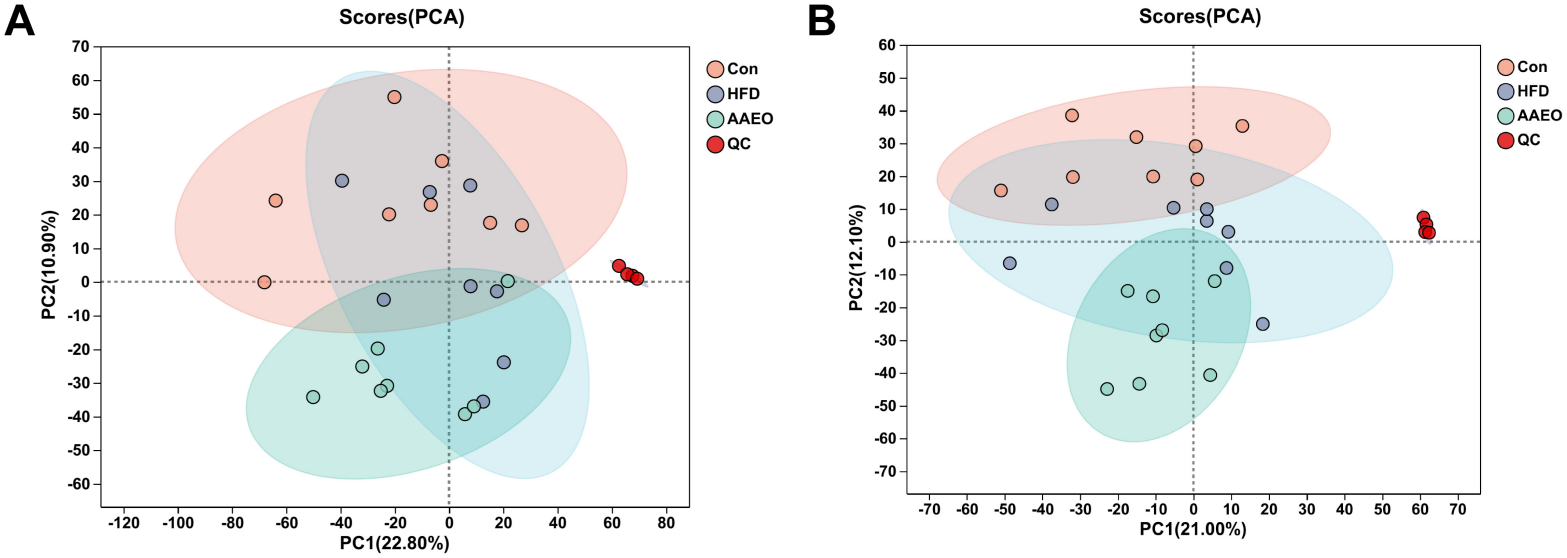
PCA score of blood of mice in each group **(A)** shows positive ions and **(B)** shows negative ions.

In order to better understand and analyze the intervention effect of AAEO on blood metabolites in mice on HFD, the blood metabolite data from each group of mice were analyzed by PCA in this study. From Figure 3, it can be seen that there was a trend of separation between the samples of mice in the HFD group and those of mice in the AAEO group regardless of the positive (Figure 3A) or negative (Figure 3B) ion mode, indicating that AAEO affected the changes of metabolites in the blood of mice. Between the mice in the Con group and the samples from the HFD group, there was a greater degree of separation within the groups, and there was a crossover between the groups, and the differences were not significant. This may be due to the fact that there were more factors that have an impact on the data, i.e., not only the dietary factors set up in this experiment, but also individual differences in mice, the stability of the instrumentation, and other external factors, resulting in the phenomenon of underfitting the data, and therefore the PLS-DA statistical method is needed to continue the analysis, and thus to ignore the random differences within the groups and to highlight the systematic differences between the groups.

**Figure 4:**
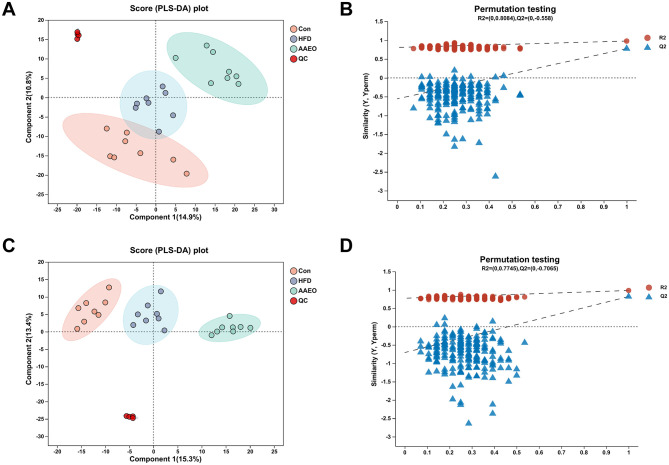
PLS-DA score map and permutation test map of blood of mice in each group **(A, B)** shows positive ions and **(C, D)** shows negative ions.

As can be shown from Figures 4A, C, there was no crossover between the samples of the Con group and HFD in either the positive or negative ion mode, and the degree of separation was good. There was no crossover or overlap between the samples of the HFD group and AAEO group. It indicated that the AAEO has a significant effect on the metabolites in the blood of mice on HFD. The replacement test graph was used to speculate whether the model was reliable or not, and the criterion for the evaluation of the replacement test was mainly the intercept of the *Q*^2^ regression line with the *Y*-axis, when the intercept was < 0.05, it indicated that the model was robust and reliable. As shown in Figures 4B, D, *Q*^2^ = −0.558 < 0.05 in the positive ion model and *Q*^2^ = −0.7065 < 0.05 in the negative ion model, indicating that the model was robust and reliable without overfitting in either ion model.

### 3.5 OPLS-DA analysis

**Figure 5:**
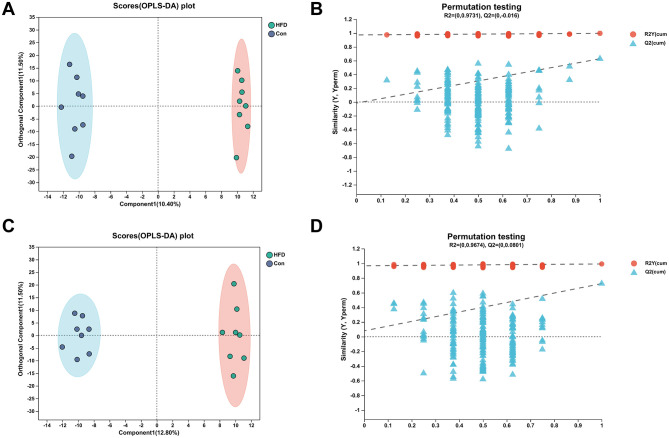
OPLS-DA model score plot and permutation test plot between Con vs. HFD **(A, B)** shows positive ions and **(C, D)** shows negative ions.

**Figure 6:**
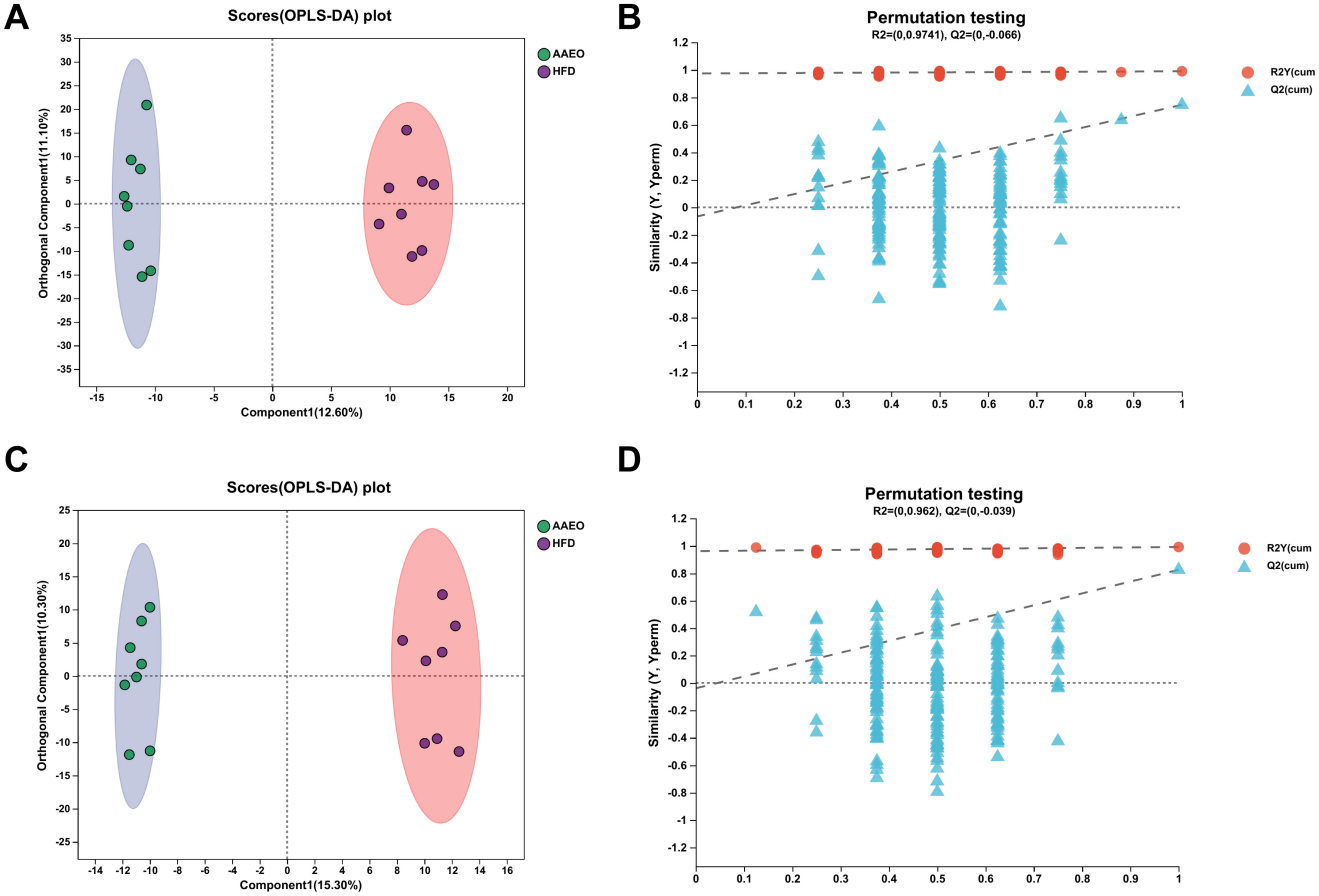
OPLS-DA model score plot and permutation test plot between HFD vs. AAEO **(A, B)** shows positive ions and **(C, D)** shows negative ions.

Orthogonal Partial Least Squares Discriminant Analysis, OPLS-DA was a derivative of PLS-DA. OPLS-DA can better distinguish between subgroups and improve the validity and parsing ability of the model. The OPLS-DA model score plot and permutation test plot between Con vs. HFD and HFD vs. AAEO were shown in Figures 5, 6A, C, respectively, and there was a clear trend of separation between Con vs. HFD and between HFD vs. AAEO in both positive and negative ion modes. And from Figures 5B, D, *Q*^2^ was < 0.05 in the positive ion mode, and *Q*^2^ was >0.05 in the negative ion mode, and with the decrease of replacement retention in the negative ion mode, *R*2 and *Q*2 decreased, and the regression line showed an upward trend, which could indicate that the replacement test was passed, and the model did not have the overfitting phenomenon. From Figures 6B, D, it could be proved that *Q*^2^ is < 0.05 in both the positive ion mode and the negative ion mode, indicating that the model was robust and reliable without overfitting in either ion mode.

The parameters of the OPLS-DA models for positive and negative ionization were listed in Table 4. The closer the *R*^2^*Y* and *Q*^2^ were to 1, the more stable and reliable the model was, *Q*^2^ > 0.5 indicated a better predictive ability of the model, and *Q*^2^ < 0.5 indicated a poorer predictive ability of the model. As can be seen from the table, the cumulative explanatory rate of *R*^2^*Y* for the comparison between the two groups in both the positive and negative ion modes exceed 0.9, indicating that at least 90% of the variance in the Y-measure can be explained by these OPLS-DA models. The *Q*^2^ calculated by cross-validation showed that the predictions of the OPLS-DA models were good for all groups. In particular, the *R*^2^*Y* and *Q*^2^ between the Con vs. HFD groups were close to 1, indicating that the HFD-induced obesity model in this study, modeling was successful. In addition, *Q*^2^ > 0.5 between the HFD vs. AAEO groups indicated that ingestion of AAEO could successfully modulate the metabolite composition caused by HFD.

**Table 4:** OPLS-DA model parameters.

**Group**	**Ion mode**	***R*^2^*X* (cum)**	***R*^2^*Y* (cum)**	***Q*^2^ (cum)**
Con vs. HFD	Positive ion	0.219	0.996	0.627
	Negative ion	0.243	0.992	0.724
HFD vs. AAEO	Positive ion	0.237	0.990	0.746
	Negative ion	0.256	0.992	0.826

*R*^2^*X* and *R*^2^*Y* denote the explanatory rates of the constructed model for the *X* and *Y* matrices, respectively, and *R*^2^*X* (cum) and *R*^2^*Y* (cum) denote the cumulative explanatory rates; *Q*^2^ denotes the predictive power of the model.

### 3.6 Screening and identification of differential metabolites between different groups

Screening was performed based on the variable importance of projection (VIP) analysis obtained from the OPLS-DA model. Metabolites with VIP value >1 and *p*-value < 0.05 were considered as significantly different metabolites between the two groups. Table 5 showed the number of significantly different metabolites from the comparative analysis between the two groups. A total of 170 significantly different metabolites were identified in the positive ion mode and 156 significantly different metabolites were identified in the negative ion mode. Compared with the Con group, there were 79 significantly different metabolites in the positive ion mode and 68 in the negative ion mode in the HFD group, and 123 significantly different metabolites in the positive ion mode and 127 in the negative ion mode in the AAEO group. Compared with HFD, there were 91 significantly different metabolites in positive ion mode and 78 significantly different metabolites in negative ion mode in the AAEO group.

**Table 5:** The number of metabolites with significant difference.

**Mode**	**Total number**	**Con^1^_vs_HFD^2^**	**HFD_vs_AAEO^3^**	**Con_vs_AAEO**
Pos	1,534 (170)	611 (79)	693 (91)	958 (123)
Neg	1,233 (156)	554 (68)	557 (78)	878 (127)

Numbers in parentheses represent the number of named differential metabolites identified. ^1^Con, control diet; ^2^HFD, high fat diet; ^3^AAEO, high fat diet with HEO.

Supplementary Table S1 showed the significantly different metabolites in the Con group compared with the HFD group screened according to VIP > 2 and *p*-value < 0.05. A total of 60 significantly different metabolites were identified, including six amino acids, peptides, and analogs, four fatty acids and conjugates, two glycerophosphocholines, two bile acids, alcohols and derivatives, one glycerophosphocholines, five carbohydrates and carbohydrate conjugates, and so on. Based on the abundance of metabolites after normalized treatment, 10 metabolites were found to be significantly up-regulated (*P* < 0.05) and 50 metabolites were found to be significantly down-regulated (*P* < 0.05) in the HFD group compared with Con group.

Supplementary Table S2 showed the significantly different metabolites in the AAEO group compared with the HFD group according to the VIP > 2 and *p*-value < 0.05 screening. A total of 79 significantly different metabolites were identified, including six Amino acids, peptides, and analogs, four fatty acids and conjugates, three fatty acyl glycosides, three carbohydrates and carbohydrate conjugates, two triterpenoids, and three terpene glycosides, and others. Based on the abundance of metabolites after normalized treatments, 22 metabolites were found to be significantly up-regulated (*P* < 0.05) and 57 metabolites were found to be significantly down-regulated (*P* < 0.05) in the AAEO group as compared to HFD group.

### 3.7 Differential metabolite analysis for multiple comparisons

**Figure 7:**
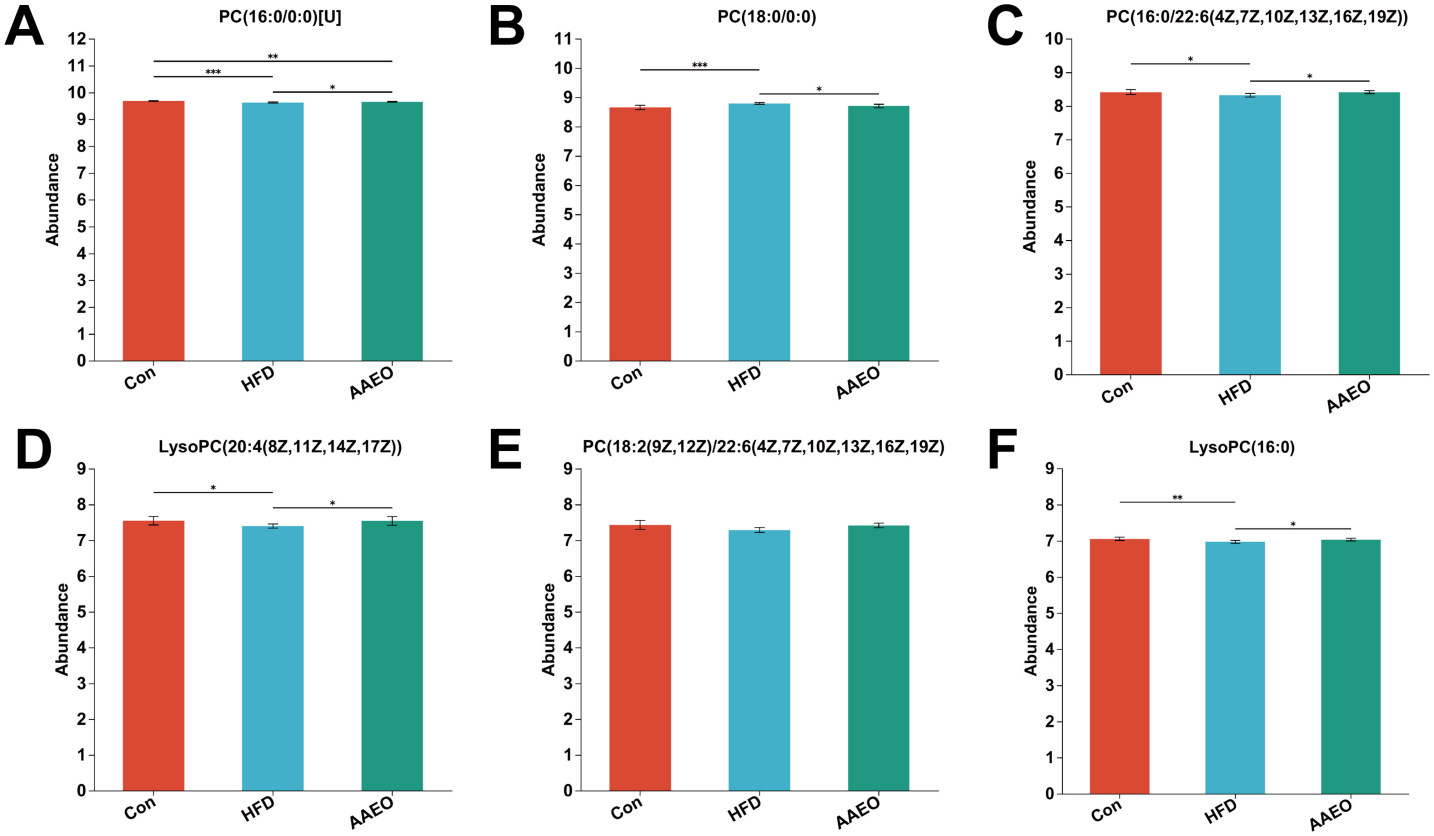
*Artemisia argyi* essential oil (AAEO) affects the main differential metabolites **(A–F)** associated with metabolic syndrome (top 20), the significance between groups represented by *was *p* < 0.05, **represented *p* < 0.01, ***represented *p* < 0.001.

The major differential metabolites of AAEO associated with obesity screened according to the Kruskal–Wallis rank sum test are shown in Figure 7. The most abundant differential metabolite was PC (16:0/0:0) [U] (Figure 7A), which was significantly lower in the HFD group than in the Con group (*P* < 0.05), and it was significantly elevated by AAEO intake (*P* < 0.05). However, the content of PC (18:0/0:0) in the HFD group was significantly higher than that in the Con group (*P* < 0.05), and the intake of AAEO significantly decreased its content (Figure 7B) (*P* < 0.05). In addition, PC [16:0/22:6(4Z,7Z,10Z,13Z,16Z,19Z)], LysoPC [20:4(8Z,11Z,14Z,17Z)], PC [18:2(9Z,12Z)/22:6(4Z,7Z,10Z,13Z,16Z,19Z)], and LysoPC (16:0) were significantly lower than those in the Con group and were restored after AAEO ingestion (*P* < 0.05) (Figures 7C–F).

### 3.8 Enrichment analysis of KEGG functional pathways for differential metabolites

**Figure 8:**
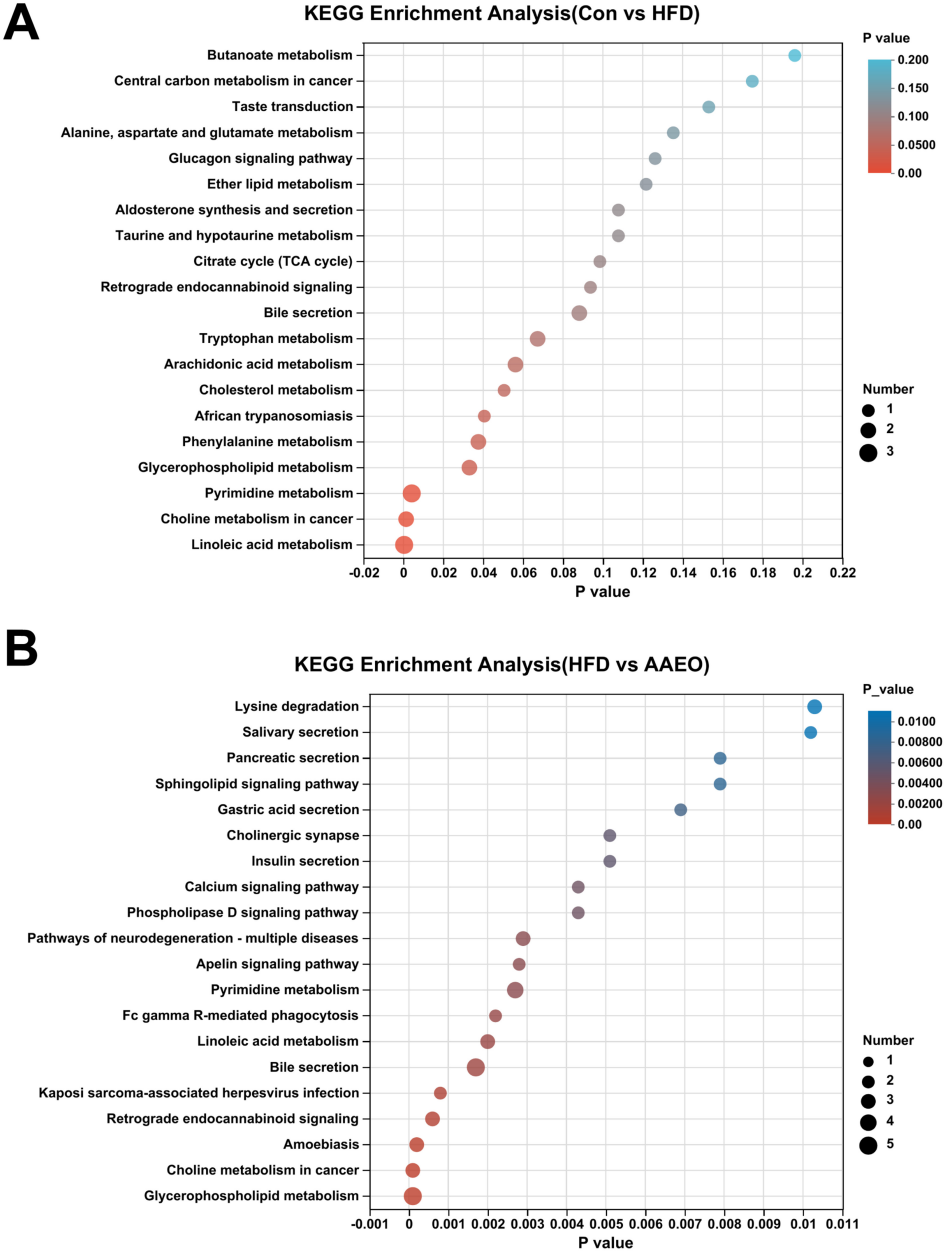
Enrichment analysis diagram of KEGG pathway in Con vs. HFD group **(A)** and enrichment analysis diagram of KEGG pathway in AAEO vs. HFD **(B)**.

Based on the difference metabolites between the two groups screened earlier, the KEGG pathway enrichment in each group was further screened by using the calculation method of num in study/num in pop. The KEGG pathway enrichment in the Con vs. HFD group and the AAEO vs HFD group was shown in Figure 8. It was shown in Figure 8A by analyzing the KEGG pathways in the Con vs. HFD group, we can obtain that there were 20 major pathways enriched. There were 20 major enriched pathways. Among them, linoleic acid metabolism, pyrimidine metabolism, glycerophospholipid metabolism and phenylalanine metabolism were significantly higher than other metabolic pathways (*P* < 0.05). In addition, some of them focused on the metabolic pathways of fat metabolism regulation, such as cholesterol metabolism, and fatty acid metabolism pathways, such as arachidonic acid metabolism. As shown in Figure 8B, there were 20 major enriched pathways by analyzing the KEGG pathways in the AAEO vs. HFD group. Glycerophospholipid metabolism had the highest enrichment rate and was significantly higher than other pathways (*P* < 0.05). This was followed by retrograde endocannabinoid signaling, bile secretion, linoleic acid metabolism, Fc gamma *R*-mediated phagocytosis, pyrimidine metabolism and apelin signaling pathway. Two of them were the main pathways involved in lipid metabolism, including glycerophospholipid metabolism and linoleic acid metabolism.

## 4 Discussion

Medicinal plants are nature's gift to human beings to help them battle against various ailments since thousands of years ago. Now with the globalization of pursuing improved quality of life, there is actually a general rising tendency in demand for dietary function plants in the world (38). A report from the World Health Organization (WHO) disclosed that almost 80% of the world's population relies on nonconventional drug-treatment, particularly of medicinal herb, in their primary healthcare (39). However, although great progress is being made in uncovering the chemistry and bioactivities of functional food plants, their exact biological functions and regulation mechanisms largely remain to be elucidated.

*Artemisia argyi* was used as a traditional remedy for various diseases in Asia, modern studies have proved its modulatory effects on the inflammation process such as rhinitis, dermatitis and arthritis, furthermore, preparation of the essential oil has been clinically prescribed for chronic bronchitis in China for years (40, 41). GC-MS analysis in this study provided detailed chemical composition of AAEO, in which several predominant chemicals including cineole, borneol, camphor, and thujone were found to possess anti-inflammatory activities according to previous reports (42, 43). These reports imply that AAEO may be associated with anti-inflammatory properties due to the presence of the above components. The key metabolic pathways identified—linoleic acid metabolism and glycerophospholipid metabolism—are critical for lipid homeostasis. Linoleic acid, an essential fatty acid, undergoes desaturation and elongation to form bioactive lipid mediators (e.g., prostaglandins) that regulate inflammation and adipocyte function (43). Our data show that AAEO normalizes linoleic acid-derived metabolites disrupted by HFD, suggesting AAEO may mitigate HFD-induced lipid dysregulation by restoring balanced production of these mediators. The chemical composition of the essential oil of *Artemisia argyi* aerial parts in the present study was not the same as that reported in previous studies. For example, eucalyptol, (-)-camphor and viridiflorol were the main volatile compo nents of *Artemisia argyi* from Zunyi City, Guizhou Province, China, moreover, the content and composition of the volatile oil of *Artemisia argyi* were various with different growing period (44). However, eucalyptol, camphor, and caryophyllene were common constituents in *Artemisia argyi* (45, 46). These differences of chemical content and composition of the essential oils might have been due to harvest time and local, climatic and seasonal factors as well as storage duration of medicinal herbs, and these differences may result in different biological activities.

High-throughput sequencing technology had many advantages. It can not only accurately analyze the structure and diversity of gut microbiota, but also further predict the gene functions and metabolic pathways of intestinal microorganisms (47, 48). The functional composition of the metagenome was predicted by PICRUSt using marker gene data and a reference genome database obtained from 16S rRNA sequencing. The results of gut microbiota showed that the microbes were mainly involved in global and overview maps, carbohydrate metabolism, amino acid metabolism and energy metabolism. According to the results of intestinal microbial function prediction, we anticipated that AAEO would not widely alter gut and fecal microbiota function.

The rapid development of metabolomics technology has become an important research tool in the fields of clinical medicine, life sciences, and food science (49, 50). Metabolomics techniques are used to study the changes in active food ingredients during food production, processing, and storage processes, and to investigate their precise mechanisms after ingestion. In addition, the application of metabolomics in analyzing interactions between different species not only provides a broad metabolic pathway, but also explains the mechanisms of microbial host interactions, playing a key role in systems biology (51). A series of open platforms currently used for metabolomics data analysis, including convenient and complete metabolomics databases such as Kyoto Encyclopedia of Genes and Genomes (KEGG), Max library database, Metabolites and Chemical Entities (METLIN) database, and Human Metabolomics Database (HMDB), provide a sustainable information sharing platform. In order to better understand and analyze the intervention effect of AAEO on serum metabolites in HFD mice. The dose-dependent efficacy of AAEO (HEO > MEO > LEO) in regulating lipid metabolism was supported by preliminary biochemical indicators data, reinforcing that the HEO group was the optimal dose for exploring the underlying mechanisms. This study conducted PCA analysis on the serum metabolite data of mice in each group. There was a clear separation trend between the control group mice and the HFD group mice samples, indicating that HFD can affect the changes in substances in mouse serum. Similarly, AAEO can also affect changes in metabolites in the serum of HFD mice. OPLS-DA can better distinguish differences between groups, improve the effectiveness and parsing ability of the model. According to PCA and PLS-DA analysis, regardless of the ion mode, it has been proven that the model is robust and reliable, and no overfitting has occurred.

This study used LC-MS/MS technology combined with multivariate statistical methods to analyze the effect of AAEO on the metabolomics of HFD induced obese mice and screened for multiple significantly different metabolites. The results indicated that AAEO can significantly regulate diet, especially metabolic abnormalities caused by HFD. 60 and 79 significantly different metabolites were screened from the HFD and AAEO groups based on VIP > 2 and *p* < 0.05, respectively. Six significantly different metabolites related to AAEO regulation of obesity were selected through multiple group comparisons, including PC (16:0/00:0), PC (18:0/0:0) PC [16:0/22:6(4Z,7Z,10Z,13Z,16Z,19Z)], LysoPC [20:4(8Z,11Z,14Z,17Z)], PC [18:2(9Z,12Z)/22:6(4Z,7Z,10Z,13Z,16Z,19Z)], and LysoPC (16:0). In addition, the pathway regulating lipid metabolism by AAEO were mainly enriched in linoleic acid metabolism and glycerophospholipid metabolism. For glycerophospholipid metabolism, the observed changes in phosphatidylcholines (PCs) and lysophosphatidylcholines (LysoPCs) are biologically meaningful: PCs are major components of cell membranes and serve as precursors for triglyceride transport, while LysoPCs are involved in lipid signaling and fatty acid uptake (52). AAEO's restoration of these metabolites [e.g., increased PC (16:0/0:0) and decreased PC (18:0/0:0) in treated mice] indicates potential modulation of membrane integrity and triglyceride trafficking, processes central to obesity-related lipid accumulation. Xu et al. found that the metabolic pathway of polysaccharides from Polygonatum cyrtonema polysaccharides intervening in obesity included glycerophospholipid metabolism, which is similar to the results of this study (52). The modulation of glycerophospholipid metabolism by AAEO likely involves the regulation of phosphatidylcholine and lysophosphatidylcholine levels, which are critical for triglyceride transport and membrane lipid homeostasis. Xu et al. (53) found through serum metabolomics analysis that patients with mild hypercholesterolemia have four potential metabolic pathways after consuming oats, namely glycerophospholipid metabolism, alanine, aspartic acid and glutamate metabolism, sphingolipid metabolism, and retinol metabolism. So, the mechanism of *Artemisia argyi* essential oil regulating obesity is related to the enrichment pathways mentioned above. In summary, the differential metabolites screened in this study are involved in the metabolic process of HFD induced obesity, which may be potential biomarkers regulating obesity. Further in-depth analysis of the metabolic mechanism of AAEO regulating obesity is expected to provide new research directions for improving obesity.

## 5 Conclusion

In summary, this study suggested that although AAEO induced selective changes in fecal microbial functions related to amino acid and lipid metabolism, while no significant shifts were observed in global microbiota composition in HFD induced obese mice, it can alter serum metabolites in ICR mice, which were mainly related to lipid metabolism. Through metabolic pathway identification, it was shown that AAEO mainly altered the linoleic acid metabolism and glycerophospholipid metabolism pathway in mice and intervened in mice lipid metabolism.

## Data Availability

The metabolomics data have been deposited in the National Genomics Data Center (NGDC) under accession number PRJCA046608: https://ngdc.cncb.ac.cn/bioproject/browse/PRJCA046608.
