# Supplementary material for: Artemisia argyi essential oil modulates lipid metabolism via linoleic acid and glycerophospholipid pathways in high-fat diet-induced obese mice
Source: Front Nutr. 2025 Oct 1;12:1650976. doi: 10.3389/fnut.2025.1650976 (PMC12520944; doi:10.3389/fnut.2025.1650976)
Supplement: Supplementary file 1 [file Table_1.docx]

**Table S1. Significantly Differential Metabolite Identification Between HFD vs Con Groups （VIP > 2）**

| **Metabolite** | **Formula** | **VIP** | **FC(HFD/Con)** | **P_value** | **HMDB Subclass** | **Regulate** |
| --- | --- | --- | --- | --- | --- | --- |
| 3-Caffeoyl-1,5-quinolactone | C16H16O8 | 4.7297 | 0.718 | 1.21E-05 | Hydroxycinnamic acids and derivatives | down |
| Fenbendazole | C15H13N3O2S | 4.3302 | 0.8072 | 0.002155 | 2-benzimidazolylcarbamic acid esters | down |
| 2,8-Dihydroxyquinoline-beta-D-glucuronide | C15H15NO8 | 4.2092 | 0.7888 | 0.001738 | Carbohydrates and carbohydrate conjugates | down |
| 8-Deoxy-11,13-dihydroxygrosheimin | C15H20O5 | 4.1973 | 0.801 | 1.94E-06 | Terpene lactones | down |
| 5-Hydroxy-6-methoxyindole glucuronide | C15H17NO8 | 4.0944 | 0.8159 | 0.000529 | Carbohydrates and carbohydrate conjugates | down |
| L-Homocysteic acid | C4H9NO5S | 3.8381 | 0.7991 | 0.003869 | Amino acids, peptides, and analogues | down |
| 6-Hydroxy-5-methoxyindole glucuronide | C15H17NO8 | 3.7536 | 0.8157 | 0.001158 | Carbohydrates and carbohydrate conjugates | down |
| 10,11-dihydro-20-trihydroxy-leukotriene B4 | C20H34O7 | 3.7173 | 0.8756 | 0.000409 | Eicosanoids | down |
| 5-hydroxy-1-(4-methoxyphenyl)-4-methylpent-1-en-3-one | C13H16O3 | 3.5738 | 0.8427 | 0.000426 | - | down |
| Australigenin | C27H42O4 | 3.5502 | 1.1391 | 7.43E-07 | _ | up |
| 3,4,5-trihydroxy-6-({2-[2,4,6-trihydroxy-3-(3-methylbut-2-en-1-yl)phenyl]acetyl}oxy)oxane-2-carboxylic acid | C19H24O11 | 3.4669 | 0.829 | 0.00426 | - | down |
| [3-(3-phenylpropanoyl)phenyl]oxidanesulfonic acid | C15H14O5S | 3.419 | 0.8303 | 0.004486 | - | down |
| Threoninyl-Serine | C7H14N2O5 | 3.3016 | 1.2252 | 0.01567 | Amino acids, peptides, and analogues | up |
| [4-(5,7-dihydroxy-4-oxo-3,4-dihydro-2H-1-benzopyran-2-yl)phenyl]oxidanesulfonic acid | C15H12O8S | 3.1165 | 0.8339 | 0.02377 | - | down |
| 6,7,4'-Trihydroxyflavanone | C15H12O5 | 3.0876 | 0.8461 | 0.04288 | - | down |
| Loquatifolin A | C39H66O18 | 3.0081 | 1.0894 | 0.000694 | Carbohydrates and carbohydrate conjugates | up |
| (3beta,22E,24R)-3-Hydroxyergosta-5,8,22-trien-7-one | C28H42O2 | 2.9428 | 0.859 | 0.004679 | Ergostane steroids | down |
| Cinncassiol C3 | C20H30O7 | 2.9232 | 0.8773 | 0.005766 | Sesquiterpenoids | down |
| Monic acid | C18H30O6 | 2.8929 | 0.8875 | 0.002383 | Fatty acids and conjugates | down |
| [4-(5,7-dihydroxy-6,8-dimethyl-4-oxo-3,4-dihydro-2H-1-benzopyran-2-yl)phenyl]oxidanesulfonic acid | C17H16O8S | 2.8742 | 0.907 | 0.000851 | - | down |
| Epinephrine glucuronide | C15H21NO9 | 2.8625 | 0.8446 | 0.04137 | Carbohydrates and carbohydrate conjugates | down |
| S-(Indolylmethylthiohydroximoyl)-L-cysteine | C13H15N3O3S | 2.8239 | 1.1808 | 0.04394 | - | up |
| Arlatin | C15H22O4 | 2.7784 | 0.9161 | 5.47E-05 | Terpene lactones | down |
| Phenylpropionylglycine | C11H13NO3 | 2.7438 | 0.9162 | 0.001636 | Amino acids, peptides, and analogues | down |
| Hippuric acid | C9H9NO3 | 2.6207 | 0.9372 | 0.000319 | Benzoic acids and derivatives | down |
| 15-cyclohexyl pentanor PGF2alpha | C21H34O5 | 2.5973 | 0.9148 | 0.008377 | - | down |
| 3,4,5-trihydroxy-6-({2-hydroxy-3-[4-hydroxy-3-(4-hydroxy-3-methylbut-2-en-1-yl)phenyl]propanoyl}oxy)oxane-2-carboxylic acid | C20H26O11 | 2.5923 | 0.9182 | 0.002397 | - | down |
| {3-[3-(2-hydroxyphenyl)propanoyl]phenyl}oxidanesulfonic acid | C15H14O6S | 2.5465 | 0.9378 | 0.000316 | - | down |
| {3-[3-(2,5-dihydroxyphenyl)-3-oxopropyl]phenyl}oxidanesulfonic acid | C15H14O7S | 2.5283 | 0.9203 | 0.000977 | - | down |
| 2-Indolecarboxylic acid | C9H7NO2 | 2.5126 | 0.9184 | 0.006234 | Indolecarboxylic acids and derivatives | down |
| Taurocholic acid | C26H45NO7S | 2.4919 | 1.0578 | 0.000749 | Bile acids, alcohols and derivatives | up |
| N-(Carbethoxyacetyl)-4-chloro-L-tryptophan | C16H17ClN2O5 | 2.4876 | 0.9259 | 0.000572 | Amino acids, peptides, and analogues | down |
| (+/-)-Equol | C15H14O3 | 2.4826 | 0.9438 | 0.000309 | - | down |
| Naringenin-7-O-beta-D-Glucuronide | C21H20O11 | 2.4219 | 0.912 | 0.04006 | - | down |
| PI(16:0/20:4(5Z,8Z,11Z,14Z)) | C45H79O13P | 2.4171 | 0.9315 | 0.002781 | Glycerophosphoinositols | down |
| 4,7,10,13,16-docosapentaenoic acid | C22H34O2 | 2.411 | 0.9274 | 0.008871 | Fatty acids and conjugates | down |
| LysoPE(16:1(9Z)/0:0) | C21H42NO7P | 2.3826 | 0.9343 | 0.001171 | Glycerophosphoethanolamines | down |
| [(1R,16Z,24E,26E,28Z)-1-hydroxy-12-[1-(4-hydroxy-3-methoxycyclohexyl)propan-2-yl]-19,30-dimethoxy-15,17,21,23,29,35-hexamethyl-2,3,10,14,20-pentaoxo-11,36-dioxa-4-azatricyclo[30.3.1.0?,?]hexatriaconta-16,24,26,28-tetraen-18-yl]oxidanesulfonic acid | C51H79NO16S | 2.3513 | 1.0634 | 0.01057 | - | up |
| PC(16:1/0:0) | C24H48NO7P | 2.331 | 0.9563 | 0.000306 | - | down |
| Austalide L | C25H32O6 | 2.3087 | 1.0756 | 0.03238 | Flavones | up |
| Siderol | C22H34O3 | 2.3029 | 0.9563 | 0.000581 | Diterpenoids | down |
| 25-Hydroxyvitamin D3-26,23-lactone | C27H40O4 | 2.2746 | 0.9447 | 0.006555 | Vitamin D and derivatives | down |
| P-Coumaraldehyde | C9H8O2 | 2.2695 | 0.9319 | 0.01655 | _ | down |
| Phenylacetylglycine | C10H11NO3 | 2.2692 | 1.0524 | 0.004692 | Amino acids, peptides, and analogues | up |
| [4-(7-hydroxy-3,4-dihydro-2H-1-benzopyran-3-yl)phenyl]oxidanesulfonic acid | C15H14O6S | 2.2345 | 0.9586 | 0.0005 | - | down |
| Cinnamoylglycine | C11H11NO3 | 2.2245 | 0.9272 | 0.0202 | Amino acids, peptides, and analogues | down |
| 3b,12a-Dihydroxy-5a-cholanoic acid | C24H40O4 | 2.2091 | 0.9359 | 0.005361 | Bile acids, alcohols and derivatives | down |
| Acetyl-DL-Valine | C7H13NO3 | 2.2041 | 1.1072 | 0.02902 | - | up |
| 3,4-Dimethyl-5-pentyl-2-furanpropanoic acid | C14H22O3 | 2.1902 | 0.9047 | 0.03245 | Fatty acids and conjugates | down |
| PC(20:4(5Z,8Z,11Z,14Z)/22:6(4Z,7Z,10Z,13Z,16Z,19Z)) | C50H80NO8P | 2.1655 | 0.9603 | 0.002761 | Glycerophosphocholines | down |
| 7,8-Dihydro-3b,6a-dihydroxy-alpha-ionol 9-glucoside | C19H34O8 | 2.153 | 0.928 | 0.03987 | Fatty acyl glycosides | down |
| 1-(2-Furyl)butan-3-one | C8H10O2 | 2.0635 | 0.927 | 0.01781 | _ | down |
| Cucurbitacin I 2-glucoside | C36H52O12 | 2.0505 | 1.0649 | 0.04558 | Steroidal glycosides | up |
| (4E,7E,10Z,13E,16E,19E)-docosa-4,7,10,13,16,19-hexaenoic acid | C22H32O2 | 2.0077 | 0.9679 | 1.72E-05 | Fatty acids and conjugates | down |
| PC(14:0/0:0) | C22H46NO7P | 2.0007 | 0.9711 | 0.000254 | Glycerophosphocholines | down |
| Hypoglycin B | C12H18N2O5 | 1.9952 | 0.9485 | 0.004947 | Amino acids, peptides, and analogues | down |
| 3,4,5-trihydroxy-6-{3-[3-(2-hydroxyphenyl)propanoyl]phenoxy}oxane-2-carboxylic acid | C21H22O9 | 1.974 | 0.9605 | 0.01226 | - | down |
| Gln Gln Tyr Phe | C28H36N6O8 | 1.9502 | 0.9582 | 0.03318 | - | down |
| Equol 4'-O-glucuronide | C21H22O9 | 1.9484 | 0.9615 | 0.003528 | - | down |
| Gentisic acid | C7H6O4 | 1.9297 | 0.9534 | 0.0001993 | Benzoic acids and derivatives | down |

**Table S2. Significantly Differential Metabolite Identification Between AAEO vs HFD Groups （VIP > 2）**

| **Metabolite** | **Formula** | **VIP_PLS-DA** | **FC(AAEO/HFD)** | **P_value** | **HMDB Subclass** | **Regulate** |
| --- | --- | --- | --- | --- | --- | --- |
| 3,4,5-trihydroxy-6-{[(2Z)-2-(phenylmethylidene)heptyl]oxy}oxane-2-carboxylic acid | C20H28O7 | 5.5438 | 1.5421 | 1.34E-10 | - | up |
| (4-ethenyl-2-methoxyphenyl)oxidanesulfonic acid | C9H10O5S | 5.3885 | 1.6584 | 1.70E-07 | - | up |
| 20alpha-Dihydroprednisone | C21H28O5 | 5.2024 | 1.5108 | 2.56E-06 | - | up |
| 7,8-Dihydro-3b,6a-dihydroxy-alpha-ionol 9-glucoside | C19H34O8 | 4.897 | 0.733 | 2.14E-07 | Fatty acyl glycosides | down |
| Cortisol | C21H30O5 | 4.8421 | 1.3427 | 6.52E-06 | Hydroxysteroids | up |
| Monic acid | C18H30O6 | 4.5971 | 0.7148 | 1.04E-07 | Fatty acids and conjugates | down |
| 3,4,5-trihydroxy-6-{2-[(1E)-3-(4-hydroxy-2-methoxyphenyl)prop-1-en-1-yl]phenoxy}oxane-2-carboxylic acid | C22H24O9 | 4.3659 | 0.7573 | 1.61E-08 | - | down |
| 10,11-dihydro-20-trihydroxy-leukotriene B4 | C20H34O7 | 4.2255 | 0.8085 | 5.17E-06 | Eicosanoids | down |
| N-(4-aminobutyl)-3-(2,4-dihydroxy-5-methoxyphenyl) prop-2-enimidic acid | C14H20N2O4 | 4.1305 | 0.6789 | 0.03097 | - | down |
| 4-Hydroxyretinoic acid | C20H28O3 | 4.0904 | 1.2577 | 2.03E-05 | Retinoids | up |
| 9,15-dioxo-11R-hydroxy-2,3,4,5-tetranor-prostan-1,20-dioic acid | C16H24O7 | 4.053 | 1.3459 | 0.000733 | - | up |
| 5-[(3-methoxyphenyl)methyl]oxolan-2-one | C12H14O3 | 4.0158 | 1.3968 | 0.000541 | - | up |
| Formyldienolone | C21H28O4 | 4.0149 | 1.2266 | 7.24E-05 | Androstane steroids | up |
| [4-(5,7-dihydroxy-6,8-dimethyl-4-oxo-3,4-dihydro-2H-1-benzopyran-2-yl)phenyl]oxidanesulfonic acid | C17H16O8S | 3.8779 | 0.7841 | 0.00049 | - | down |
| 16-Hydroxy-3-oxo-12-oleanen-28-oic acid | C30H46O4 | 3.8011 | 0.766 | 0.002737 | Triterpenoids | down |
| Cohumulone | C20H28O5 | 3.7279 | 0.7813 | 0.000666 | Alcohols and polyols | down |
| Tetranor-PGFM | C16H26O7 | 3.7149 | 1.2449 | 0.001316 | Eicosanoids | up |
| Alpha-Amylcinnamyl formate | C15H20O2 | 3.7007 | 1.2849 | 0.000235 | _ | up |
| 3-(3,5-dihydroxyphenyl)-1-propanoic acid sulphate | C9H10O7S | 3.5798 | 0.6704 | 0.02238 | Benzenediols | down |
| Deoxyloganic acid | C16H24O9 | 3.5362 | 1.3618 | 0.006679 | Terpene glycosides | up |
| Lansic acid | C30H46O4 | 3.5059 | 0.7874 | 0.01145 | Monoterpenoids | down |
| Medicagenic acid | C30H46O6 | 3.4784 | 0.8196 | 0.00449 | Triterpenoids | down |
| Tryptophyl-Gamma-glutamate | C16H20N4O4 | 3.2562 | 1.2938 | 0.005214 | Amino acids, peptides, and analogues | up |
| {3-[3-(2-hydroxyphenyl)propanoyl]phenyl}oxidanesulfonic acid | C15H14O6S | 3.2337 | 0.8814 | 3.57E-05 | - | down |
| 3,7,8,15-Scirpenetetrol | C15H22O6 | 3.2122 | 1.2494 | 0.01058 | Sesquiterpenoids | up |
| 5-hydroxy-1-(4-methoxyphenyl)-4-methylpent-1-en-3-one | C13H16O3 | 3.2064 | 0.7976 | 0.003189 | - | down |
| Cyclopassifloic acid D | C30H48O6 | 3.2042 | 0.8492 | 0.002037 | Hybrid peptides | down |
| Prenyl arabinosyl-(1->6)-glucoside | C16H28O10 | 3.1773 | 1.283 | 0.01292 | Fatty acyl glycosides | up |
| 3,4,5-trihydroxy-6-{2-[3-(3-hydroxyphenyl)-3-oxopropyl]phenoxy}oxane-2-carboxylic acid | C21H22O9 | 3.1675 | 0.8842 | 6.98E-05 | - | down |
| {4-[(1E)-3-(4-hydroxy-2-methoxyphenyl)prop-1-en-1-yl]phenyl}oxidanesulfonic acid | C16H16O6S | 3.1327 | 0.8706 | 0.001276 | - | down |
| Arlatin | C15H22O4 | 3.123 | 0.8601 | 7.88E-05 | Terpene lactones | down |
| N-(Carbethoxyacetyl)-4-chloro-L-tryptophan | C16H17ClN2O5 | 3.0713 | 0.8535 | 0.000838 | Amino acids, peptides, and analogues | down |
| Cinncassiol C3 | C20H30O7 | 3.0422 | 0.8383 | 0.000811 | Sesquiterpenoids | down |
| Dethiobiotin | C10H18N2O3 | 2.998 | 0.7999 | 0.008356 | Fatty acids and conjugates | down |
| Glucosyl 6-hydroxy-2,6-dimethyl-2E,7-octadienoate | C16H26O8 | 2.9614 | 1.1854 | 0.007229 | Carbohydrates and carbohydrate conjugates | up |
| (+/-)-Equol | C15H14O3 | 2.9411 | 0.9058 | 6.47E-05 | - | down |
| Wogonin | C16H12O5 | 2.9321 | 0.8867 | 0.005366 | - | down |
| 3,4-Dimethyl-5-pentyl-2-furanpropanoic acid | C14H22O3 | 2.8389 | 0.8447 | 0.001452 | Fatty acids and conjugates | down |
| 2-Hydroxyestrone-1-S-glutathione | C28H37N3O9S | 2.7874 | 0.8826 | 0.01453 | Amino acids, peptides, and analogues | down |
| 6-(4-{3-[2,4-dihydroxy-5-(3-methylbut-2-en-1-yl)phenyl]-2-hydroxy-3-oxopropyl}-2-(3-methylbut-2-en-1-yl)phenoxy)-3,4,5-trihydroxyoxane-2-carboxylic acid | C31H38O11 | 2.7216 | 0.8704 | 0.01177 | - | down |
| L-2-Amino-3-methylenehexanoic acid | C7H13NO2 | 2.7042 | 0.8774 | 0.004607 | Amino acids, peptides, and analogues | down |
| Rebaudioside C | C44H70O22 | 2.6906 | 1.1128 | 0.009439 | Terpene glycosides | up |
| 6-[(2Z)-2-carboxy-2-(phenylmethylidene)ethoxy]-3,4,5-trihydroxyoxane-2-carboxylic acid | C16H18O9 | 2.6728 | 1.2538 | 0.03411 | - | up |
| Gly Phe | C11H14N2O3 | 2.6718 | 1.1412 | 0.01692 | - | up |
| (1R,3S,4S,6R)-6,9-Dihydroxyfenchone 6-O-b-D-glucoside | C16H26O8 | 2.653 | 1.1802 | 0.02545 | Terpene glycosides | up |
| ISOKOBUSONE | C14H22O2 | 2.6164 | 0.9152 | 0.00017 | Alcohols and polyols | down |
| Estrone | C18H22O2 | 2.5888 | 0.8532 | 0.02811 | Sulfated steroids | down |
| Demethyltexasin | C15H10O5 | 2.5806 | 0.8671 | 0.03129 | Isoflav-2-enes | down |
| (3S,7E,9S)-9-Hydroxy-4,7-megastigmadien-3-one 9-glucoside | C19H30O7 | 2.5765 | 0.8929 | 0.005988 | Fatty acyl glycosides | down |
| 4-Guanidinobutanoic acid | C5H11N3O2 | 2.571 | 0.8879 | 0.002003 | Amino acids, peptides, and analogues | down |
| 3,4,5-trihydroxy-6-{3-[3-(2-hydroxyphenyl)propanoyl]phenoxy}oxane-2-carboxylic acid | C21H22O9 | 2.5535 | 0.9331 | 0.000326 | - | down |
| 5,7-dihydroxy-2-phenyl-8-[3,4,5-trihydroxy-6-(hydroxymethyl)oxan-2-yl]-4H-chromen-4-one | C21H20O9 | 2.5474 | 0.8285 | 0.02709 | - | down |
| 16-bromo-9E-hexadecenoic acid | C16H29BrO2 | 2.5427 | 0.8719 | 0.0258 | - | down |
| O-Desmethylangolensin | C15H14O4 | 2.5233 | 0.817 | 0.04804 | _ | down |
| [4-(7-hydroxy-3,4-dihydro-2H-1-benzopyran-3-yl)phenyl]oxidanesulfonic acid | C15H14O6S | 2.519 | 0.9389 | 0.000117 | - | down |
| 6-Hydroxykynurenic acid | C10H7NO4 | 2.453 | 0.9172 | 0.000698 | Quinoline carboxylic acids | down |
| Ile Cys Glu Asn | C18H31N5O8S | 2.4316 | 0.9048 | 0.01292 | - | down |
| 6-{3-[3-(2,5-dihydroxyphenyl)-3-oxopropyl]phenoxy}-3,4,5-trihydroxyoxane-2-carboxylic acid | C21H22O10 | 2.4243 | 0.8843 | 0.03011 | - | down |
| {2-hydroxy-5-[3-(2-hydroxyphenyl)propanoyl]phenyl}oxidanesulfonic acid | C15H14O7S | 2.4024 | 0.8831 | 0.01632 | - | down |
| 3'-N'-Acetylfusarochromanone | C17H22N2O5 | 2.4004 | 0.8948 | 0.006109 | - | down |
| 3,4,5-trihydroxy-6-{3-[2-hydroxy-3-(2,4,6-trihydroxyphenyl)propyl]phenoxy}oxane-2-carboxylic acid | C21H24O11 | 2.3127 | 0.9186 | 0.001982 | - | down |
| Equol 4'-O-glucuronide | C21H22O9 | 2.2603 | 0.945 | 0.000157 | - | down |
| Pyrogallol-2-O-glucuronide | C12H14O9 | 2.2583 | 0.8902 | 0.02393 | Carbohydrates and carbohydrate conjugates | down |
| 2,3-Dinor-TXB2 | C18H30O6 | 2.2547 | 0.8822 | 0.009252 | Eicosanoids | down |
| 1alpha-Hydroxyarbusculin A | C15H22O4 | 2.2226 | 0.9004 | 0.008606 | Terpene lactones | down |
| Val His Cys Phe | C23H32N6O5S | 2.1518 | 1.0735 | 0.02803 | - | up |
| Chrysophanol | C15H10O4 | 2.0587 | 0.9309 | 0.04536 | Anthraquinones | down |
| Traumatic Acid | C12H20O4 | 2.0438 | 0.9309 | 0.001174 | Fatty acids and conjugates | down |
| 3,7-DIHYDROXYFLAVONE | C15H10O4 | 2.0208 | 0.9148 | 0.04753 | - | down |
| Fluvoxamino acid | C14H17F3N2O3 | 2.0114 | 0.9418 | 0.001391 | Trifluoromethylbenzenes | down |
| Phenyllactic acid | C9H10O3 | 2.0084 | 0.933 | 0.006785 | _ | down |
| Ser Ile Ala Asp | C16H28N4O8 | 2.0066 | 0.9554 | 0.0005 | - | down |
| Asparaginyl-Proline | C9H15N3O4 | 2.0065 | 0.9589 | 6.78E-06 | Amino acids, peptides, and analogues | down |
| Xi-2,3-Dihydro-2-oxo-1H-indole-3-acetic acid | C10H9NO3 | 2.0045 | 0.942 | 0.008868 | Indolyl carboxylic acids and derivatives | down |
| P-[N-Propyl-N-(3-hydroxypropyl)sulfamoyl]benzoic acid | C13H19NO5S | 1.9934 | 0.9426 | 0.0007784 | - | down |
| (2E)-3-[3-(sulfooxy)phenyl]prop-2-enoic acid | C9H8O6S | 1.9716 | 0.9542 | 0.0003652 | Hydroxycinnamic acids and derivatives | down |
| Phenylalanyl-Serine | C12H16N2O4 | 1.9661 | 1.074 | 0.03458 | Amino acids, peptides, and analogues | up |
| (E)-Casimiroedine | C21H27N3O6 | 1.9626 | 1.0643 | 0.02452 | Carbohydrates and carbohydrate conjugates | up |
| 3-Indolepropionic acid | C11H11NO2 | 1.9413 | 0.927 | 0.02727 | Indolyl carboxylic acids and derivatives | down |
